# Do community scorecards improve utilisation of health services in community clinics: experience from a rural area of Bangladesh

**DOI:** 10.1186/s12939-020-01266-5

**Published:** 2020-11-02

**Authors:** S. M. A. Hanifi, Aazia Hossain, Asiful Haidar Chowdhury, Shahidul Hoque, Mohammad Abdus Selim, Shehrin Shaila Mahmood, Abbas Bhuiya

**Affiliations:** 1 Health Systems and Population Studies Division, Universal Health Coverage, icddr,b. 68 Shaheed Tajuddin Ahmed Sarani, Mohakhali, Dhaka, 1212 Bangladesh; 2 IMPACT study, ARK Foundation, Gulshan, Dhaka, 1212 Bangladesh; 3 School of Health Sciences and Social Work, University of Portsmouth, Portsmouth, UK

**Keywords:** Community scorecard, Community participation, Community clinic, Service utilisation

## Abstract

**Background:**

The government of Bangladesh initiated community clinics (CC) to extend the reach of public health services and these facilities were planned to be run through community participation. However, utilisation of CC services is still very low. Evidence indicates community score card is an effective tool to increase utilisation of services from health facility through regular interface meeting between service providers and beneficiary. We investigated whether community scorecards (CSC) improve utilisation of health services provided by CCs in rural area of Bangladesh.

**Methods:**

This study was conducted from December 2017 to November 2018. Three intervention and three control CCs were selected from Chakaria, a rural sub-district of Bangladesh. CSC was introduced with the Community Groups and Community Support Groups in intervention CCs between January to October 2018. Data were collected through observation of CCs during operational hours, key informant interviews, focus group discussions, and from DHIS2. Utilisation of CC services was compared between intervention and control areas, pre and post CSC intervention.

**Results:**

Post CSC intervention, community awareness about CC services, utilisation of clinic operational hours, and accountability of healthcare providers have increased in the intervention CCs. Utilisation of primary healthcare services including family planning services, antenatal care, postnatal care and basic health services have significantly improved in intervention CCs.

**Conclusion:**

CSC is an effective tool to increase the service utilization provided by CCs by ensuring community awareness and participation, and service providers’ accountability. Policy makers and concerned authorities may take necessary steps to integrate community scorecard in the health system by incorporating it in CCs.

## Background

The Government of Bangladesh introduced community clinics (CCs) in the 1990s with an aim to deliver primary health care, family planning and nutrition services to rural people at the grassroots level. Currently there are 13,500 CCs in Bangladesh, aimed to cover every 6000 rural population [1]. The CCs deliver “Essential Service Package” to the rural people from a fixed community-based centre [2]. The services provided by CCs include maternal and neonatal health services, Integrated Management of Childhood Illness (IMCI), reproductive health and family planning services, Expanded Programme on Immunisation (EPI), nutritional education and provision of micro-nutrient supplements, provision of health education and counseling, screening of chronic non communicable diseases, treatment of minor ailments, common diseases and first aid, and establishing referral linkage with other facilities [1].

CC set up is a unique example of public-private partnership as it runs through community participation. Each CC is managed by a Community Group (CG) and a Community Support Group (CSG) [3]. The CG is a 13 to 17 member managing body of the CC which takes care of CC’s day to day operations, monitoring and evaluation of CC’s performance and community participation, coordination with stakeholders and fund generation [3, 4]. Each CG is headed by the local government representative (*union parishad* member) and is designed to have active participation of local community members, local government representatives which includes men, women, elderly, adolescent girls and boys, and representatives from the poorest asset quintiles. The members of this group are supposed to be trained on clinic management by local health authorities prior to clinics being opened. To supplement the CG, each CC has 3 CSGs comprising of 13–17 members each with one third female members [4]. The local government representatives are included in the management committee. Apart from helping the CG in managing the clinic, the CSG works to raise awareness about the CC and its services in the community [4].

CC services are provided by domiciliary workers from two wings of the Ministry of Health and Family Welfare (MOHFW) - the Directorate General of Health Services (DGHS) and the Directorate General of Family Planning (DGFP) [5]. The CCs are manned by one Community Health Care Provider (CHCP), one Family Welfare Assistant (FWA) and one Health Assistant (HA), all of whom are members of the CG [3]. Under the government directive, CHCPs are mandated to work full time at the CC, while the FWAs and HA are mandated to work part time (3 days a week) [5].CHCPs are appointed by the DGHS and they serve as the member secretary of the CG and their responsibilities are to provide range of preventive and primary curative care services, to manage the clinic and mobilise people to avail the services provided by the CCs [2, 5]. The FWAs are appointed by DGFP and are responsible for providing community-based family planning services. The HA are responsible for providing immunisation and other primary health care services under the DGHS [5].

Despite the widespread establishment of CCs that aim to bring healthcare to the doorsteps of the rural population, utilisation of services remains low. Utilisation of antenatal (ANC) care from service provider of CC was 1.6 and 5.2% from CHCP and HA/FWA respectively [6]. Most CCs face shortage of scheduled staff, as well as irregular attendance of providers [7, 8]. Further, lack of properly defined roles and responsibilities of different staff and poor accountability of the providers hinder optimal service provision at the CCs. Only 8% of the CCs have documented quality assurance procedures and client feedback is low. Poor provider behaviour towards patients is another common complaint that prevents patients from seeking healthcare at CCs. Inconvenient location of CCs, the lack of adequate physical infrastructure of the facility, and lack logistics are further impediments. The Bangladesh Health Facility Survey 2014 reported that most CCs lack emergency transport, communication equipment, computers and internet connections. Only a fifth of CCs had electric connection with the national electric grid, and only 9% of the CC had regular electricity [8]. Shortage of supplied medicine and their inefficient use by CC staff add to these challenges. In addition, the absence of active participation from community in healthcare delivery critically restricts efficient use of CCs and their available resources [8]. The formation of the two community clinic management groups, the CGs and the CSGs, as mentioned above were originally aimed at ensuring equity, accountability and quality [9]. However, formations of the CGs and support groups have so far been slow and lack community ownership. Stakeholders are not properly aware of CC’s purposes and there is weak communication and low involvement of local government institution. These challenges contribute towards the low utilisation of CC services. Thus a mechanism to sensitize the CC committee groups and the community needs to be in place to improve the functionality of the CCs.

Community engagement in the management of healthcare delivery and local level planning has been seen as a successful strategy to improve health service delivery and increase utilisation [10, 11]. Strong cohesion of community and social network have also helped to improve healthcare delivery and utilisation of health services in similar settings [12, 13]. Social accountability approaches such as citizen report cards, village health committees and community score cards (CSC) have helped in increasing accountability in health facility monitoring [14]. The CSC, a social audit tool that brings together service users and providers of a particular service to assess, plan, monitor and evaluate services, in particular has worked successfully in improving healthcare utilisation in rural settings in other developing countries such as Afghanistan, Democratic Republic of Congo and Malawi [15–17]. These social accountability approaches have been found to induce intermediate changes like improved transparency, more efficient use of resources, empowered citizens, improved perceptions of health services, which results in better health outcome [18].

To complement the monitoring mechanism of CCs and to improve their functionality, the Future Health Systems (FHS) Bangladesh team at icddr,b (International Centre for Diarrhoeal Disease Research, Bangladesh) implemented a community scorecard (CSC) to ensure community participation and provider accountability in three CCs in Chakaria, Cox’s Bazar. In Bangladesh, this was the first time CSCs were applied to the health service delivery system. This paper aims to measure the effect of CSC on utilisation of basic health services provided by CCs in Chakaria, a rural sub-district of Bangladesh.

## Methods

### Study area

**Fig. 1:**
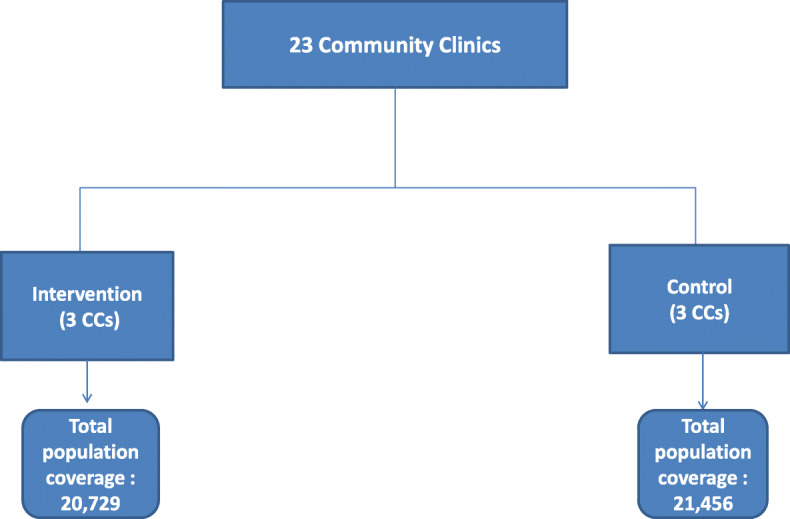
Intervention and control CCs and their population coverage (under Chakaria HDSS)

The intervention was carried out in the Chakaria *upazila* (sub-district) of Cox’s Bazar district under the Chattogram division where icddr,b has been running a Health and Demographic Surveillance System (HDSS) since 1999. The HDSS covers twenty three CCs in 49 villages consisting of 17,000 households and 85,000 populations. Information on health and demographic indicators and records of vital events of all the households, including healthcare seeking behavior, are collected through quarterly household visits. CSC was implemented in three CCs in Manikpur, Baraitali and Shaharbil unions of Chakaria. These three clinics are referred to as “intervention CCs”. Three other CCs (in Harbhang, Dhemoshia and B.M.Char unions) were selected as control CCs. The intervention and control CCs were selected purposively, considering similar catchment area, set up, logistics and keeping in mind that the two types of CCs do not fall in adjacent villages to avoid spillover effect. The total population coverage of the three intervention clinics is 20,729 with an average of 6909 people per CC. For the three control CCs, the total population coverage is 21,456 with an average of 7152 people per CC (Fig. 1).

Apart from CCs in the catchment areas, there are other private and public health service facilities that also provide services to the community. The presence of NGOs, public and private services in both intervention and control areas is similar (Additional file 1).

### Description of CSC intervention

The CSC intervention activities in the intervention CCs can be categorised into four basic phases:

Phase 1- Planning and preparation: All planned activities were listed out. Rapport building activities were done with CC committees (CG and CSG); orientation sessions were held to clarify the functions of CCs, the roles and responsibilities of CG and CSG members, and to introduce the CSC; stakeholder and accountability mapping sessions were done with community and providers.

Phase 2- Community and provider generated scorecard: For this current study, CGs were taken to represent the provider group, and the CSGs were taken to be the community representative group. Separate sessions were held with community and providers where problems in the CCs and their related indicators were identified, listed, scored (on a scale of 1 to 5, 5 being the highest score) and prioritised.

Phase 3 - Interface meeting and action plan setting: Consensus was built between community and providers around common sets of priority indicators and targets; and action plans were developed with responsible persons, timeline, and required resources to attain those set targets.

Phase 4 - Action plan implementation and monitoring and evaluation: Responsible persons took steps to carry out set tasks and monitor progress of CSC implementation.

One implementation cycle constituted of activities from phase two to four which were then repeated for the next cycle. The time gap between each cycle of CSC was 2 months. Between January to October of 2018, three cycles of CSC were completed.

In each cycle, three provider (CG) meetings, and nine community (CSG) meetings were scheduled to be held. However, in the 2nd cycle, one CSG meeting could not be held due to water logging of area in Baraitali during monsoon season. In the CG meetings, there were a total of 35 participants in Cycle 1, 36 in Cycle 2, and 34 in Cycle 3. In the CSG meetings, there were 121 participants in Cycle 1, 102 in Cycle 2, and 124 in Cycle 3. In addition, in each cycle, three interface meetings (phase 3) were also held where representatives from both CG and CSG participated (Table 1).

**Table 1:** Number of meetings and participants of CSC intervention across meeting type and cycle

Cycle	CG		CSG		Interface		Total	
	No. of meeting	No. of participants	No. of meeting	No. of participants	No. of meeting	No. of participants	No. of meeting	No. of participants
**1st**	3	35	9	121	3	57	15	213
**2nd**	3	36	8	102	3	64	14	202
**3rd**	3	34	9	124	3	70	15	228
**Total**	**9**	**105**	**26**	**347**	**9**	**191**	**44**	**643**

To study the trend in utilisation of health services, the intervention and controls CCs were observed for 5 days before CSC implementation and for 5 days after three cycles of CSC implementation.

### Data collection

**Fig. 2:**
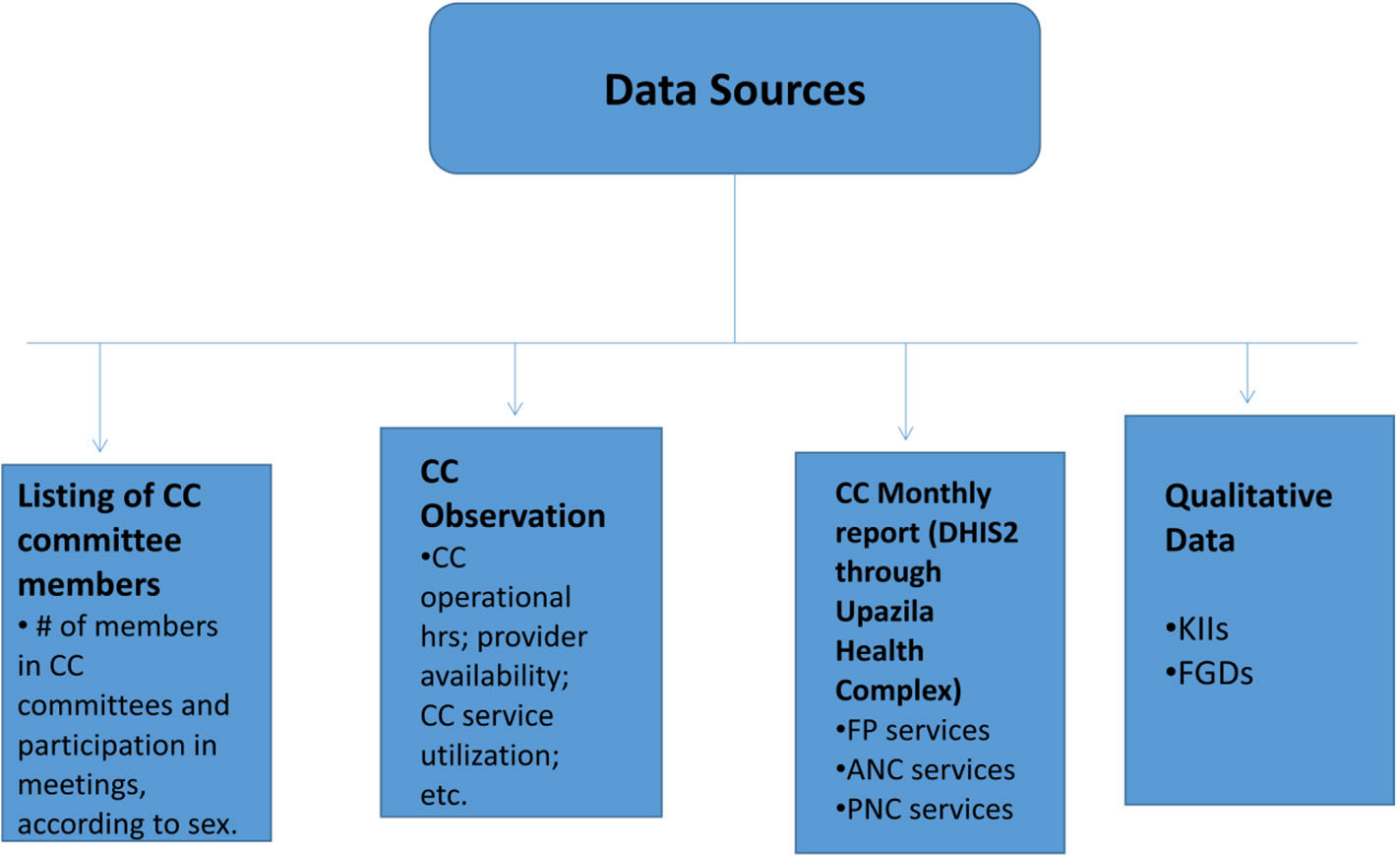
Data Sources

Data for this paper were collected from four sources (Fig. 2) described below.

### Listing of CC committee members

FHS team members identified the CC committee members (CG and CSG), listed the members according to sex. In addition, in all the meetings, number of participants was also recorded according to their sex (Table 2).

**Table 2:** Formation of CC committees and participation in CC meetings

Meeting type	Formation of committee			Participation in meetings				
	Female	Male	Female/Male ratio	No. of meetings	Female	Male	Total participants	Female/Male ratio
**CG**	54	96	0.56	9	26	79	105	0.33
**CSG**	117	307	0.38	26	50	297	347	0.17

### Observation and document review at CC

Data was also collected through observation during full service delivery hour (from 9 am to 3 pm) at each of the six (intervention and control) CCs for 5 days before (December 2017 to January 2018) and 5 days after three cycles of CSC interventions (November 2018). Facility observation was done using a standard questionnaire (Additional file 2), combining both qualitative and quantitative approaches and by using pre-approved guidelines by the Institutional Review Board (IRB) of icddr,b. The observers were three experienced staff of icddr,b, which including a field research manager with background in public health, a senior field research officer with sociology background, and a field research supervisor with psychology background. Alongside observation, relevant documents were reviewed and the following information were collected through observation: CC operational hours; availability of designated service providers; availability of medicine to be stocked in shelves as per protocol; service utilisation statistics data of under 5 children, and adult females and adult males; provision and availability of services and medical equipment; status of facility premises, information desk, registration and waiting area, outpatient department (OPD), infection prevention practices; functionality of CC committees health education, cleanliness of facility, etcetera.

#### DHIS2

Data on utilisation of family planning services, antenatal care services and post natal care (PNC) services at intervention and control CCs were also collected from the CC Monthly Reports, obtained through the Chakaria Upazila Health Complex from DHIS2.

### Qualitative data

To understand community perception about CSC and its role in improving quality of service and service utilisation at CC, the project team conducted a qualitative survey in the catchment area of six study CCs of Chakaria (three intervention and three control). Qualitative data were collected through key informant interviews (KII) and focus group discussions (FGD) with various stakeholders representing beneficiaries, CG and CSG members, healthcare providers and local government officials. The investigators of the study developed the guidelines for KIIs and FGDs which were approved by icddr,b’s IRB. A two member team of qualitative researchers, with background in anthropology, were oriented by the Principal Investigator (PI) and Co-PI to carry out the qualitative data collection. A total of twenty seven KIIs (twenty in the intervention clinics and seven in control CCs) and six FGDs (four in intervention CC and two in control CCs) were done in November 2018, after CSC intervention. KIIs were carried out one to one with CG and CSG members (CHCP, Union Parishad Member, Member Secretary, land owner, influential community people, well off, poor member from CSG, and adolescent member from CSG) of intervention and control CCs. Key informants were purposively selected from each category within the CG/CSG groups so that it was representative of all members. The six FGDs, with an average of six participants per discussion, were carried out with CG and CSG members of intervention and control CCs. FGDs were conducted by a three member team from icddr,b (moderator, note taker and observer), which included two qualitative researchers.

### Indicators

Table 3 presents the indicators and tools used for data collection. We used seven indicators to measure the impact of CSC (Table 3). To measure the effect of CSC on health service utilisation, we did not include childhood immunisation as an indicator, as some of the selected CCs do not offer EPI services.

**Table 3:** Indicators used to measure utilisation of CC

Indicators	Tools used
1. Regular CC meetings	CC observation; KII; FGD
2. Availability of CC staff at scheduled operation hours	CC observation; KII; FGD
3. Operational hours of CC	CC observation
4. Healthcare service sought from CC	CC observation
5. Family planning services received	CC Monthly report (DHIS2 through Upazila Health Complex)
6. Receiving ANC	CC Monthly report (DHIS2 through Upazila Health Complex)
7. Receiving PNC	CC Monthly report (DHIS2 through Upazila Health Complex)

### Data analysis

#### Duration of service delivery

The utilisation of service hours were calculated by dividing observed hours of service provision by scheduled operational hours of the CCs. Comparison was made between the intervention and control CCs.

#### Service utilisation through CC observation

Percentage of service utilisation from CC observation for under-five children, adult females, adult male clients were calculated by dividing the number of clients of respective group who received the services by the estimated number of population in the respective groups of CC catchment area.

#### Service utilisation through DHIS2

Contraceptive prevalence rate was calculated by number of women who received contraceptives (condom, injectables, oral pills) from CC divided by total number of eligible women (currently married women between 15 and 49 years of age) in the CC catchment area. Percentage of ANC visits was calculated by number of ANC received by pregnant women divided by total number of pregnant women in the CC catchment area. Percentage of PNC visits was calculated by dividing the number of PNC received by mothers with newborns in the CC catchment area.

Quantitative data were analysed using STATA 14.

For qualitative data, thematic, content analysis was done. Themes for qualitative analysis were determined from concerned issues, indicators of the CC e.g. service hours of the CC, types of services provided, accountability and behavior of the providers. Findings obtained from quantitative data were elaborated and supported by relevant quotes from qualitative data.

## Results

### Community awareness

Lack of awareness and functionality amongst the CC committees regarding the clinic, and the members’ roles and responsibilities in management was observed in control CCs:

*“We don’t really have regular meetings. They just take our signatures. The CHCP who is responsible, she’s called a few meetings … I think we’ve had maybe 1 or 2 meetings in the past 4 years. No one comes to the meetings. I think no one is interested in the meetings. We haven’t really discussed much when we met, anyway. We did have one meeting in the beginning but there was no discussion about how to improve the CC”* (KII 1)

Providers were also unaware about their specific roles and responsibilities and have forgotten about their terms of reference that is listed in CC manual:

*“I’ve forgotten the contents of the (CC) manual. We were trained/oriented (with the manual) a long time ago. I’ve forgotten it.* (KII 7)

FGD participants and key informants at the intervention CCs stated that through the process of CSC implementation, community became more aware about the community clinics and the services offered by these clinics:

*“Before CSC, we did not know much (about CC). We knew there was a health facility here, but whether they provided quality healthcare services; whether they have medicines … we did not know all this. After attending the CSC meetings, we found out that this clinic has machines to check blood sugar and blood pressure. They have thermometers so patients can check their body temperature in case of fever. On top of that, they disburse medicines for fever, diarrhoea and other medicines. We don’t have to go to Chakaria (Upazila Health Complex) for everything, we can avail quality treatment at the community clinic. I myself have learnt this and have also taken steps to raise awareness amongst the community about the clinic as well.”(FGD 2)*

*“Many people didn’t go to the community clinics earlier. The fact that there’s a community clinic here, they offer so many services, treatment is available, they offer delivery facilities, disburse medicines; provide check-ups … .no one really knew all this that well. But now, after the community scorecard, every knows and are more aware”* (FGD 1)

*“There is a (community) clinic here. We, the general people of the community, were not aware of the services that are available at this facility. If someone became unwell, they would rush to the Upazila (Health Complex) to seek treatment, especially people from the middle class and lower class. We have invited them and held monthly meetings with them. Now the people in the locality are more aware”* (KII 5)

### CC operational hours and provider accountability

**Fig. 3:**
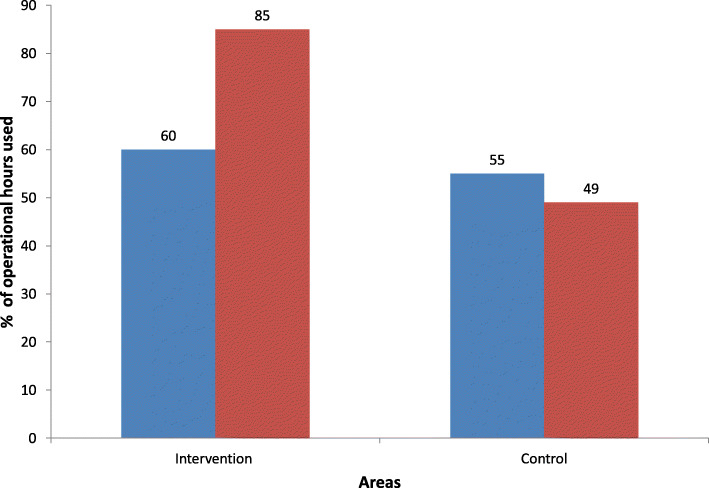
Operational hours utilised in intervention and control areas, pre and post CSC implementation. *Pre* *Post >*

On average, all three intervention clinics were operating for longer hours than control clinics post CSC implementation. Operational hours at intervention CCs increased from 60% pre CSC to 86% post CSC (*p* < 0.01), whereas in control CCs, the operational hours remained similar (50% versus 54%, *p* = 0.61). (Table 4 and Fig. 3).

**Table 4:** Utilisation of CC operational hours

Indicator	Intervention CC		Control CC	
	Pre	Post	Pre	Post
No. of days observed	13	15	12	15
Scheduled operational hours	78	90	72	90
Service provided (hours)	47	77	39	45
Operational hours utilised (%)	60	86	54	50

Findings from KIIs also supported this result and shows that the CSC has increased provider accountability in terms of their availability at scheduled hours, as well as helping beneficiaries become more aware about CC operational hours:

*“There was no timetable at the CC earlier, which has now been established because of the scorecard. A notice with CC operational time is hung up at the premises now. If the CHCP is not available at the CC, maybe s/he has a meeting somewhere … then s/he has to write that on the notice board in advance. When people see the notice board, they will not ask further questions. They now know why the clinic is closed or why the provider is not available. Earlier they didn’t understand, now they know”* (KII 9)

The community also perceived that the CSC has helped in improving provider accountability and their behavior towards patients:

*“We used to sit in the CG meetings and discuss all these issues … we would tell the community what we discussed as well. We talked about improving many issues (through the scorecard) … logistics and maintenance of the CC, their (healthcare provider’s) behavior and attitude, whether the CC opens and closes at the designated times, whether proper healthcare and medicines are available and being provided, whether there are any problems in availing services. Many of these problems have been solved … many things. A lot of (positive) change has come in the attitude and behavior of the provider (towards patients)* (KII 5).

However in the control CC, key informants stated that that there was lack of accountability and participation from the providers in terms of attending meetings and carrying out responsibilities:

*“The member, as in the president, he knows his responsibilities. But he does not follow his responsibilities; he never comes to the meetings.”* (KII 7)

### Utilisation of primary healthcare services

**Fig. 4:**
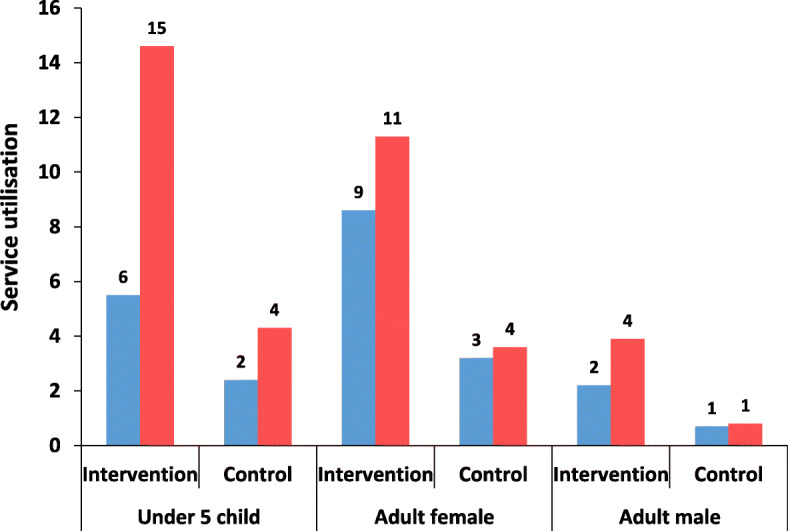
Service utilisation of intervention and control CC pre and post intervention. *Pre* *Post >*

For all groups of users (under 5 children, adult females, and adult males) utilisation of healthcare services increased from pre CSC to post CSC, for intervention CCs compared to control CCs (*p* < 0.05). In case of control CCs, significant increase was seen only for children less than 5 years of age, but the increase was higher for intervention CCs (2.64 [95% CI 2.14–3.27]) than control (1.81 [95% CI 1.30–2.54) (Table 5 and Fig. 4).

**Table 5:** Service utilisation of intervention and control CC pre and post intervention

Type of CC	Under 5 Children			Adult Females			Adult Males		
	Pre	Post	***p***- value*	Pre	Post	***p***-value*	Pre	Post	***p***-value*
**Intervention**	5.5 (134/2425)	14.6 (358/2452)	0.01	8.6 (459/5328)	11.3 (610/5387)	0.05	2.2 (114/5119)	3.9 (203/5176)	0.01
**Control**	2.4 (59/2510)	4.3 (108/2538)	0.01	3.2 (178/5515)	3.6 (200/5576)	0.31	0.7 (38/5299)	0.8 (42/5357)	0.69

*p-value for the test of no difference between and after intervention

The changes in utilisation of specific services in intervention and control CCs, pre and post CSC implementation (Table 6). In the intervention clinics, the percentage of eligible women who received family planning services increased from 2% pre intervention to 8%, post intervention (*p* < 0.01). For ANC visits, the percentage of pregnant women who availed ANC services from intervention CCs increased from 18% (pre CSC) to 22% (post CSC) (*p* < 0.01). The percentage of new mothers with babies born within 6 weeks, who availed PNC services at the intervention CCs increased from 5% (pre CSC) to 9% (post CSC) (*p* < 0.01).

**Table 6:** Type of services provided in control and intervention CC, pre and post CSC implementation

Service Type		Intervention CC		Control CC	
		Pre CSC (%)	Post CSC (%)	Pre CSC (%)	Post CSC (%)
**Family Planning**	Condom	0.03	0.18	0.18	0.18
	Injectables	0.11	1.18	1.23	1.26
	Oral pill	1.77	6.26	1.52	1.32
	All	1.91	7.61	2.94	2.76
	**Total eligible women**	**9948**	**10,127**	**10,296**	**10,481**
**ANC visits**	1st ANC	5.81	8.06	6.37	5.86
	2nd ANC	4.89	5.36	3.06	3.05
	3rd ANC	4.64	5.41	2.51	2.41
	4th ANC or above	2.60	2.80	1.35	0.98
	All	17.94	21.63	13.28	12.31
	**Total number of pregnant women**	**1962**	**1997**	**1995**	**2031**
**PNC visits**	1st PNC	0.6	1.1	2.26	2.76
	2nd PNC	0.97	2.08	1.47	1.44
	3rd PNC	2.7	5.37	0.49	0.30
	All	5.10	8.55	4.22	4.50
	**Total number of women who delivered babies within 6 weeks**	**1609**	**1638**	**1636**	**1665**

In contrast, the utilisation of services remained similar in the control CCs, pre and post intervention. There was no significant change (*p* = 0.54) in eligible women availing family planning services; the change in pregnant women availing ANC was also not significant after implementation of CSC (*p* = 0.34); and change in mothers with newborns availing PNC was negligible (*p* = 0.67) (Table 6).

Findings from the qualitative survey also support the result that CSC helped in increasing utilisation of CC services. Key informants in intervention CCs stated:

*“We get more patients now than we did earlier. Every day, we treat over 50 patients. Yesterday we had 46 general patients and 5 children. A year/ 2 years ago, we would get a maximum of 25 to 30 patients per day. Now there is a lot more awareness, icddr,b has helped (through its CSC process). That is why utilization has increased.”* (KII 13)

*“The scorecard definitely has an impact here. People gradually got to know about the CC as the committees got more involved and active and raised awareness. Now that everyone knows about the clinics, the number of patients has also increased”* (KII 12)

## Discussion

### Main findings

Although service utilisation at CCs is very low, introducing CSC in CCs increased community awareness about CC services and participation in CC committee meetings. The CSC has also resulted in increased provider accountability and has boosted CC operational hours and increased utilisation of basic health services.

### Consistency with previous studies

Various methods have been tested to improve quality and equity in health service provision at local level around the developing world. Among these, community engagement in planning, managing and financial oversight has proved to sustain gains achieved in some of the health programmes [19–21]. Studies in Afghanistan, Democratic Republic of Congo and Malawi found that CSCs have facilitated in increasing community participation and awareness, accountability of providers and utilisation of services [15, 16, 22]. The findings in this paper, that active community participation has a positive effect on improving community awareness and provider accountability and thus enhance utilisation of health services are also consistent with other studies [11, 12] in the study area. Findings from the study regarding service utilisation rate in intervention and control CCs at baseline are consistent with findings from a study on assessment of CCs supported by the World Health Organisation [23].

### Interpretation

CSC, as a tool between providers and beneficiaries, is a pathway to improve health service utilisation at community clinics by improving community awareness and ownership, and increasing provider accountability.

The CSC brought about positive changes in terms of community awareness about community clinics and its services. Before CSC implementation, most community members viewed the CCs are mere drug dispensaries. They were not aware about the various services offered at CCs, about the CC management committees and the responsibilities of the providers. In all three intervention clinics, through the CSC process, the CC committees became more active, they held more regular meetings, and were more pro-active in mobilising the community and thus community awareness about CCs increased. The finding is consistent with a study conducted among women in Bangladesh that show that increased community awareness leads to increased utilisation of health services [24].

It is assumed that the increase in operational hours after CSC implementation at the intervention CCs is a result of the CSC which helped to mobilise both CG and CSG and in specific, increased provider accountability. Providers are more aware about their roles and accountable towards their responsibilities at the CCs. In the intervention CCs, post CSC implementation, signboards have been hung up stating the available services and drugs at the facilities, the opening and closing hours, holidays, availability of CHCP, etc. This, in turn, helped to increase community awareness and utilisation of CC services.

Even accounting for the increase, the fact still remains that overall health service utilisation at CCs is still very low. An important issue is to explore why more than 80% of the community choose to seek healthcare from informal healthcare provider (IHPs) (who are not formally trained and do not have medical qualifications) over qualified providers such as the CHCPs at CCs. CCs provide free treatment and free prescribed medication for its beneficiaries. On the other hand, IHPs charge money for medicines, so it raises the question as to why IHPs are so popular. In terms of operational hours, CCs are only open till 3 pm whereas patients can visit IHPs till late at night. It is important to understand whether the short operational hours of the CC are inconvenient and acting as a barrier for the community to access its services.

The increase in utilisation of basic services could partly be a result of increased community awareness and increased provider accountability at the CCs. In addition, the CSC helped in identifying and solving problems faced by both users and providers and made the CCs better equipped, which could also have led to the increase in service utilisation. Comparison between the intervention and control CCs show that there have been significant increases in utilisation of family planning services, ANC of pregnant women, and PNC services of new mothers. Additionally, CC observation shows that utilisation of health services of children less than 5 years, adult females and adult males also increased significantly post CSC for intervention clinics.

Even though CCs were established to reach the rural grassroots population, the inverse healthcare law stands true in this case as well. A recent study shows that awareness about CC services is higher among the rich and educated community people [24]. It is crucial to investigate who is using the CC services more – the better off or the disadvantaged groups. It is important that the poor members of the community can access and utilise CC services. Mobilising the CG and CSG, making sure that all segments of community are properly represented can help in increasing voice of the disadvantaged groups in CC management. CSC tool can play a vital role in increasing service utilisation of CC amongst the poor.

### Strengths and weaknesses

For a holistic approach, we used both quantitative and qualitative data to understand the effect of CSC on the community’s usage of services offered at CCs. The study findings show that community participation and accountability of health sector actors increase in presence of CSC. Even though the study was done in a small area and over a short duration, improvements have been seen in utilisation of health services at intervention CCs post CSC implementation compared to control CCs. This increase could have been more but was thwarted by the absence of the community healthcare provider (CHCP) in one of the intervention CCs, who was on maternity leave partly during the intervention.

## Conclusion

Although this study only had three CCs in control and three in intervention and it ran for a short period of time, CSC intervention brought positive changes in community awareness, ownership, provider accountability and CC service utilisation. If scaled up at a national level, the impact of CSC can be enormous and can help in increasing utilisation for millions of Bangladeshis. A large scale study can look in details at the effectiveness and impact of CSC for increasing utilisation of health service, especially among the poor and help in attaining universal health coverage and reaching for the Sustainable Development Goals.

## Supplementary information

**Additional file 1.** Health service facilities at catchment area of CCs.

**Additional file 2.** Community Clinic Observation checklist.

## Data Availability

Data and supporting material will be available following the Data Sharing Policy of icddr,b. All requests for data should be made to the corresponding author at hanifi@icddrb.org

## Abbreviations

CC: Community Clinic
CSC: Community scorecard
CSG: Community Support Group
CG: Community Group
IRB: Institutional Review Board
PI: Principal Investigator
KII: Key Informant Interview
FGD: Focus Group Discussion
CHCP: Community Health Care Provider
HA: Health Assistant
FWA: Family Welfare Assistant
DHIS2: District Health Information System, version 2
icddr,b: International Centre for Diarrhoeal Disease Research, Bangladesh
ANC: Antenatal care
PNC: Postnatal care
IMCI: Integrated Management of Childhood Illness
EPI: Expanded Programme on Immunization
FHS: Future Health Systems
HDSS: Health and Demographic System
NGO: Non government organization
FDSR: Family Development Services and Research
OPD: Outpatient department
IHP: Informal healthcare provider
DGHS: Directorate General of Health Services
DGFP: Directorate General of Family Planning
MoHFW: Ministry of Health and Family Welfare
