# Supplementary material for: Do community scorecards improve utilisation of health services in community clinics: experience from a rural area of Bangladesh
Source: Int J Equity Health. 2020 Nov 2;19:149. doi: 10.1186/s12939-020-01266-5 (PMC7604960; doi:10.1186/s12939-020-01266-5)
Supplement: Supplementary file 1 — Additional file 1. Health service facilities at catchment area of CCs. [file 12939_2020_1266_MOESM1_ESM.docx]

| **Category of health facilities** | **Health Facility** | **Catchment area of Intervention CCs** | **Catchment area of Control CCs** |
| --- | --- | --- | --- |
| NGO | BRAC |  |  |
|  | FDSR |  | x |
| Public | UHFWC | x |  |
|  | CC |  |  |
| Private | IHP/Pharmacy |  |  |
|  | Homeopathic |  |  |

**Additional file 1: Health service facilities at catchment area of CCs**
