# Supplementary material for: Do community scorecards improve utilisation of health services in community clinics: experience from a rural area of Bangladesh
Source: Int J Equity Health. 2020 Nov 2;19:149. doi: 10.1186/s12939-020-01266-5 (PMC7604960; doi:10.1186/s12939-020-01266-5)
Supplement: Supplementary file 2 — Additional file 2. Community Clinic Observation checklist. [file 12939_2020_1266_MOESM2_ESM.docx]

**Additional file 2 – Community Clinic Observation checklist**

| Date of visit: |  |
| --- | --- |
| Name of the visiting health facility: |  |

Address of facility:

Village/Mohalla: Union/ Ward:

Upazila/Area:

| Key Informant Health service Provider(s) | | |
| --- | --- | --- |
| Sl. No. | Name | Designation |
| 1 |  |  |

| **Sl. No.** | **A. Issues** | | | **B. Information from health facility observation** | | | | | | |
| --- | --- | --- | --- | --- | --- | --- | --- | --- | --- | --- |
| **1** | **Service delivery hour** | | | Facility record | | | | Observation | | |
|  |  |  |  |  | | | |  | | |
| **2** | **Human resources** | | | a. Designation | b. Recruited # | | | c. Present # | d. Absent # | e. Reasons of absence |
|  |  | | |  |  | | |  |  |  |
|  |  | | |  |  | | |  |  |  |
|  |  | | |  |  | | |  |  |  |
|  |  | | |  |  | | |  |  |  |
|  | | | | | | | | | | |
| **3** | **# of Patients attended at different time point of the day** | | | | | | | | | |
|  |  | | | a. Adult | | | | b. Under 5 | | |
|  | Time points | | | M | | F | | M | | F |
|  | 9-10 a.m. | | |  | |  | |  | |  |
|  | 10-11 a.m. | | |  | |  | |  | |  |
|  | 11 a.m. -12 p.m. | | |  | |  | |  | |  |
|  | 12 – 1 p.m. | | |  | |  | |  | |  |
|  | 1-2 p.m. | | |  | |  | |  | |  |
|  | 2-3 p.m. | | |  | |  | |  | |  |
|  | 3-4 p.m. | | |  | |  | |  | |  |
|  | | | | | | | | | | |
| **4** | **Provision and availability of services** | | | | | | | | | |
|  |  | | a. Provision | | | | b. Available | | c. Not Available | |
|  | a. | |  | | | |  | |  | |
|  | b. | |  | | | |  | |  | |
|  | c. | |  | | | |  | |  | |
|  |  | | | | | | | | | |
| **5** | **Provision and availability of medicines/ FP materials** | | | | | | | | | |
|  |  | a. Provision | | | | | b. Available | | c. Not Available | |
|  | a. |  | | | | |  | |  | |
|  | b. |  | | | | |  | |  | |
|  | c. |  | | | | |  | |  | |
|  | d. |  | | | | |  | |  | |

| **6** | **Provision and availability of Medical Equipment** | | | |
| --- | --- | --- | --- | --- |
|  |  | a. Provision | b. Available | c. Not Available |
|  | a. |  |  |  |
|  | b. |  |  |  |
|  | c. |  |  |  |
|  | d. |  |  |  |

|  |  | **Yes** | **No** | **Partial** | **Comments** |
| --- | --- | --- | --- | --- | --- |
| **7** | **Facility Premises** | | | | |
| a. | Facility signboard is visible from outside |  |  |  |  |
| b. | Facility building has posters of behavior change communication (BCC) materials only |  |  |  |  |
| c. | Facility premise is free from Commercial banners |  |  |  |  |
| d. | Display board with information on available medicine list is present |  |  |  |  |
| e. | Facility opening hours are displayed at facility entrance |  |  |  |  |
| f. | Water supply/tube well is available |  |  |  |  |
| g. | Facility has adequate fan and lighting |  |  |  |  |
|  | | | | | |
| **8** | **Information desk, registration and waiting area** | | | | |
| a. | Complaint/suggestion boxes are available and easily accessible |  |  |  |  |
| b | Safe drinking water is available for patients and visitors |  |  |  |  |
| c. | Registers are updated regularly |  |  |  |  |
| d. | Have separate waiting area |  |  |  |  |
|  | | | | | |
| **9** | **Outpatient department (OPD)** | | | | |
| a. | All rooms/sections are labeled |  |  |  |  |
| b. | Adequate light and ventilation in examination rooms |  |  |  |  |
| c. | Examination bed is present in CHCP’s room |  |  |  |  |
| d. | Examination bed has clean linen |  |  |  |  |
| e. | Examination bed has bedside screen for privacy |  |  |  |  |
| f. | Stairs for patients to climb on bed is available |  |  |  |  |
| g. | Waste bins are placed in right place |  |  |  |  |
| h. | Body of waste bins are clean |  |  |  |  |
| i. | Pharmacy department/section is labeled |  |  |  |  |
|  |  |  |  |  |  |
| **10** | **Infection prevention practices** | | | | |
| a. | Use of chlorine solution |  |  |  |  |
| b. | Sterile equipment used |  |  |  |  |
| c. | Gloves used |  |  |  |  |
| d. | Hand washing facility available |  |  |  |  |
|  | | | | | |
| **11** | **CC committee** | | | | |
| a. | CC Committee (CG, CSG) register is up to date |  |  |  |  |
| b. | CG committee meeting schedule available |  |  |  |  |
| c. | CG committee meetings held regularly |  |  |  |  |
| d. | CSG committee meeting schedule available |  |  |  |  |
| e. | CSG committee meetings held regularly |  |  |  |  |
|  |  |  |  |  |  |
| **12** | **Health Education** |  |  |  |  |
| a. | Routine health education sessions for health education available |  |  |  |  |
| b. | Routine sessions are conducted on time regularly |  |  |  |  |
|  |  |  |  |  |  |
| **13** | **Cleanliness of facility** | **Score (1 to 5; 1=poor, 5=Best)** | | | **Comments** |
| a. | CC premise is free from Garbage |  | | |  |
| b. | Adequate seating arrangement in waiting area available |  | | |  |
| c. | Cleanliness of facility Walls |  | | |  |
| d. | Cleanliness of facility floors |  | | |  |
| e. | Cleanliness of facility ceiling |  | | |  |
| f. | Cleanliness of facility windows |  | | |  |
| g. | Cleanliness of facility doors |  | | |  |
|  |  |  |  |  |  |
| **14** | **Treatment procedure (observation)** | | | | |
| a. | Provider are dressed appropriately |  | | |  |
| b. | Privacy of patients maintained |  | | |  |
| c. | Behaviors of provider appropriate to patients |  | | |  |
| d. | Medicines were distributed properly |  | | |  |
|  |  |  |  |  |  |
|  |  |  |  |  |  |

| Name of observer |  |
| --- | --- |
| Visit ending time |  |

| 5 | **Provision and availability of medicines/ FP Materials** | | | |
| --- | --- | --- | --- | --- |
|  |  | a. Porvision | b. Available | c. Not Available |
|  | **5.1** | Albendazole tablet 400mg (chewable) |  |  |
|  | **5.2** | Antacid tablet 650mg (chewable) |  |  |
|  | **5.3** | Calcium Lactate Tablet 300mg |  |  |
|  | **5.4** | Chlorpheniramine Tablet 4mg |  |  |
|  | **5.5** | Co-trimoxazole tablet 120mg |  |  |
|  | **5.6** | Co-trimoxazole tablet 960mg |  |  |
|  | **5.7** | Ferrouse Fumarate and Folic Acid Tablet (200.40mg) |  |  |
|  | **5.8** | hyoscine Butylbromide Tablet 10 mg |  |  |
|  | **5.9** | Metronidazole Tablet 400mg |  |  |
|  | **5.10** | Paracetamol Suspension (120mg/5ml) 60ml |  |  |
|  | **5.11** | Paracetamol Tablet 500mg |  |  |
|  | **5.12** | Penicillin V Tablet 250mg |  |  |
|  | **5.13** | Salbutamol Tablet 2mg |  |  |
|  | **5.14** | Vitamin-A- Capsule 200000 IU |  |  |
|  | **5.15** | Vitamin-B-Complex Tablet |  |  |
|  | **5.16** | Zinc Dispersible Tablet 20 mg |  |  |
|  | **5.17** | Amoxicillin Dry Syrup (125mg/5ml) 100ml |  |  |
|  | **5.18** | Amoxicillin Peadiatric drop (125mg/1.25ml) 15ml |  |  |
|  | **5.19** | Benzyl Benzoate Application (25% W/V) 100ml |  |  |
|  | **5.20** | Chlorpheniramine Maleate Syrup (2mg/5ml) 60ml |  |  |
|  | **5.21** | Salbutamol Syrup (2mg/ 5 ml) 60ml |  |  |
|  | **5.22** | Amoxicillin Capsule 250mg |  |  |
|  | **5.23** | Chloramphenicol Eye Drop 0.5%, 10 ml |  |  |
|  | **5.24** | Compound Benzoic Acid Ointment 1.0kg |  |  |
|  | **5.25** | (Benzoic Acid and Salicylic Acid Ointment) |  |  |
|  | **5.26** | Gentian Violet Topical Solution 2%10ml |  |  |
|  | **5.27** | Neomycin and Bacitracin Ointment 10g |  |  |
|  | **5.28** | Oral Rehydration Salt (ORS) |  |  |
|  | **5.29** | Chloramphenicol eye ointment |  |  |
|  | **5.30** | Doxycycnine Cap (100mg) |  |  |
|  | **5.31** | GentianViolet Solution |  |  |
|  | **5.32** | Tetna |  |  |
|  | **5.33** | Keto |  |  |
|  | **5.34** | Condom |  |  |
|  | **5.35** | Injection |  |  |
|  | **5.36** | Pill |  |  |
|  |  |  |  |  |
|  |  |  |  |  |
